# Supplementary material for: Stakeholder perspectives on a patient-centred intervention (DIALOG+) for adolescents with common mental disorders in Colombia: A qualitative study
Source: PLoS One. 2022 Aug 11;17(8):e0272066. doi: 10.1371/journal.pone.0272066 (PMC9371287; doi:10.1371/journal.pone.0272066)
Supplement: S1 File — (DOCX) [file pone.0272066.s001.docx]

# **Supporting Information**

## **Topic guide**

Prior to the OFG session participants and their parents/guardians must send their signed informed consent and sociodemographic questionnaire to the study coordinator.

### **Welcome and ground rules**

Introduction from the moderator and researchers.

The moderator will inform participants that the session will be video and audio recorded and will ask them if they agree to activate their cameras. Additionally, the moderator should reiterate the confidentiality and security of data obtained.

Highlight the importance of their contributions and that there are no right or wrong answers.

Ask participants to avoid speaking at the same time and remind them that they can leave or take a break whenever they want.

-Consent verification

Participants have their informed consent prior to the OFG session. However, during the OFG session the moderator will read it out loud and participants will give their verbal consent additionally to the written one. This process will be recorded.

-Initial question

The moderator will ask participants to introduce themselves.

### **Introduction to the DIALOG+ intervention and app and purpose of the OFG**

We will discuss your opinions and insights on the DIALOG+ intervention and your suggestions on how we could adapt it to the Colombian adolescent population.

Share the aim of the DIALOG + intervention

Using the DIALOG+ app the moderator will explain the eleven life domains, the rating system, the four-step approach, and actions or tasks.

**S1 Table 1.** DIALOG+ Domains.

| Domain | Description |
| --- | --- |
| Mental health | *Symptoms, emotional state, coping, well-being* |
| Physical health | *Maintaining health, diet, physical activity, any health conditions* |
| Job situation | *Employment/unemployment status, seeking work* |
| Accommodation | *Living situation* |
| Leisure activities | *Sports, hobbies, other activities during free time* |
| Partner/family | *Relationship with partner/family members* |
| Friendships | *Relationships with friends* |
| Personal safety | *Risk of harm from self-and/or others* |
| Medication | *Is the medication working, adherence, side-effects?* |
| Practical help | *Resources available to support the patient and help them complete actions between sessions and improve situation* |
| Meetings | *Meetings with the clinician* |

During this OFG we aim to obtain your honest comments on the DIALOG+ intervention and App. We would like to know what you like or dislike and if you consider that any changes or modifications are necessary in order to make it more suitable for adolescents.

1. [General impression]

Overall, what are your impressions on the DIALOG+ intervention?

- Likes/dislikes
- Do you think it can be useful to adolescents with depression and/or anxiety?

1. [Exploring concerns of adolescents]

In your opinion, what do you think are the main concerns of adolescents?

What do you think are the main things that affect the mental health and wellbeing of adolescents?

1. [Classification scale]

What do you think of the eleven different domains that we evaluate with the DIALOG+ intervention?

And what are your opinions on the way they are evaluated? (1 to 7 rating system)

- Likes/dislikes
- Do you consider that these areas are relevant/adequate for adolescents?
- How do you think that they could be improved?
- Are there missing domains for this population?
- Do you think there are domains that could be eliminated?

1. [Choosing domains]

What do you think of choosing domains in order to keep discussing them?

- How many domains do you consider are adequate to keep discussing?
- Are these domains easy to discuss in depth with adolescents?

1. [Four-step approach]

- What did you like or dislike of the four-step approach?
- Do you understand what the resources mean?
- Do you consider it would be easy to identify and suggest resources to the patient?
- What are your thoughts the task approach?
- How can this approach be improved?

1. [Barriers and facilitators]

Is there something that can make the use of DIALOG+ easier or difficult during a consultation?

- What could make it easier?
- What could make it harder? What are the barriers?
- Is there enough time in a traditional consultation?

1. [Impact on consultations]

How do you think that the use of a tablet and the intervention could affect the doctor/patient relationship?

- Would the discussion of these topics make the experience different?
- How? Why?
- If yes, how would these changes make you feel?

1. If you had to choose just one change for the DIALOG+ intervention, what would it be?
2. Do you know any adolescents with anxiety or depression that would like to participate in our project?

### **Closure**

Thank participants for their time. Answer any additional questions.

Stop the recording of the session.

End OFG.
